# Immunocontraceptives: New Approaches to Fertility Control

**DOI:** 10.1155/2014/868196

**Published:** 2014-07-10

**Authors:** Kiranjeet Kaur, Vijay Prabha

**Affiliations:** Department of Microbiology, Panjab University, Chandigarh 160014, India

## Abstract

The rapidly increasing global population has bowed the attention of family planning and associated reproductive health programmes in the direction of providing a safe and reliable method which can be used to limit family size. The world population is estimated to exceed a phenomenal 10 billion by the year 2050 A.D., thus presenting a real jeopardy of overpopulation with severe implications for the future. Despite the availability of contraceptive methods, there are over one million elective abortions globally each year due to unintended pregnancies, having devastating impact on reproductive health of women worldwide. This highlights the need for the development of newer and improved contraceptive methods. A novel contraceptive approach that is gaining substantial attention is “immunocontraception” targeting gamete production, gamete outcome, or gamete function. Amongst these, use of sperm antigens (gamete function) seems to be an exciting and feasible approach. However, the variability of immune response and time lag to attain titer among vaccinated individuals after active immunization has highlighted the potential relevance of preformed antibodies in this league. This review is an attempt to analyze the current status and progress of immunocontraceptive approaches with respect to their establishment as a future fertility control agent.

## 1. Introduction

The world population has exceeded 6.43 × 10^9^ and is increasing by 1 × 10^9^ every 12 years [1]. Besides population explosion, unintended pregnancies and elective abortions are a major public health issue. This overgrowth will also worsen the impact of many social, ecological, economical, and medical trends [2]. With the serious global issue of overpopulation, especially in developing countries, it becomes a necessity to find an effective way to control the rapid growth of population.

Contraception is an accepted route for the control of population explosion in the world [2]. The need for contraception varies for different couples from postponing child bearing, spacing childbirth and limiting family size to absolute no child bearing which is highly personal decision based on their individual preferences, medical history, life style and other factors [3]. The characteristics of an ideal contraceptive include acceptability of user, no loss of natural feeling, no side effects, relatively inexpensive, effective, easy to use and reversible. Contraceptive choices are accessible in case of both men and women. Currently practiced contraceptive options available for men include condoms, withdrawal and vasectomy [4, 5]. In case of females, the contraceptive methods available worldwide include oral contraceptive (OC) pills, injectable contraceptives, contraceptive implants, intrauterine devices, contraceptive vaginal ring, barrier methods, surgical sterilization, spermicides and natural family planning methods.

A novel contraceptive approach that is gaining substantial attention is immunocontraception, which is the use of contraceptive vaccines (CVs) or preformed antibodies to prevent fertilization. As with the conventional vaccines, CVs utilise the body's defense system to wedge an essential step in the reproductive process [6]. A successful contraceptive vaccine must meet a number of criteria; it must be reliable, easy to administer, safe, affordable, widely acceptable, and capable of evoking homogeneous response and must bestow a high level of contraceptive efficacy. There are three major categories involved in the development of CVs including gamete production, namely, gonadotropin releasing hormone (GnRH), follicle stimulating hormone (FSH), and luteinizing hormone (LH); gamete outcome, namely, human chorionic gonadotropin (hCG), or gamete function, namely, zona pellucida (ZP) and sperm antigens [7]. Of all, validation and practicability for the development of antisperm vaccines targeting prefertilization events seem to be much more encouraging and promising. In this article we have tried to review the current status of the immunocontraceptive approaches and discussed their relative merits as a future contraceptive.

## 2. Immunocontraception

The advent of “immunocontraception” represents the first truly novel approach to the development of family planning methods. It involves the administration of a vaccine that induces an adaptive immune response which causes an animal to become temporarily infertile [8]. Immunocontraception promises many advantages over the methods of contraception currently available for human use which include high target specificity, long term action but not permanent, relatively inexpensive, lack of endocrine or metabolic side effects, and easy to use and does not require insertion of an implant or device and does not require surgical intervention [9].

**Figure 1:**
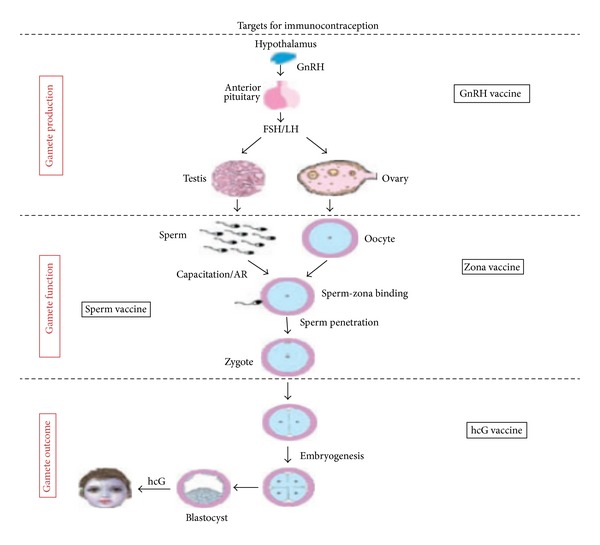
Schematic model indicating various targets that are being explored for the contraceptive vaccine development. These include targeting gamete production [gonadotropin-releasing hormone (GnRH), follicle-stimulating hormone (FSH), and luteinizing hormone (LH)], gamete function [zona pellucida (ZP) proteins of the oocytes and sperm antigens], and gamete outcome (human chorionic gonadotropin [hCG]) (adapted from [9]).

Currently, there are three major categories involved in the development of immunocontraception including gamete production, gamete outcome, or gamete function [7] (Figure 1).

### 2.1. Gamete Production

Gamete production includes the synthesis of reproductive cells from gonads, that is, sperm from testes and ovum from ovaries. The first hormone in the chain is GnRH. It regulates the release of two peptide hormones from anterior pituitary, namely, FSH and LH, collectively called gonadotropins. Immunoneutralization of any of these hormones can prevent the binding of respective hormone with their receptor which may result in failure of conception.

#### 2.1.1. Gonadotropin Releasing Hormone (GnRH)

The decapeptide GnRH stimulates the release of the gonadotropins and is present both in males and females; thereby a vaccine against GnRH is feasible in both sexes. The hypothalamic GnRH-I (also referred to as GnRH or luteinizing hormone releasing hormone (LHRH)) is clearly the main hormone in the hypothalamic-pituitary-gonadal axis, besides a possible direct role of this decapeptide in extrapituitary organs, such as testis, prostate, and placenta [10]. The gene coding for GnRH-I is located on chromosome 8p21-p11.2.

When GnRH vaccine was tested on human subjects, including breastfeeding women by The National Institute of Immunology, India, and the Population Control Council, USA, the vaccine essentially resulted in castration and halted testosterone production [11]. However, these results were accompanied with impotence and loss of body hair in case of men and affected menopause in women [12]. Hence, an analogue of GnRH was prepared by linking with different carriers in an attempt to make improved vaccine [7]. Different carriers, namely, diphtheria toxoid (DT), tetanus toxoid (TT),* Mycobacterium tuberculosis* hsp 70 [13], thioredoxin [14], keyhole limpet hemocyanin [15], and ovalbumin, were employed. It was observed that the conjugation with any of the carriers induced specific antibody response. However, even after multiple injections, low titer antibodies were generated. It is well known that conjugation of protein/peptide with appropriate adjuvant can augment the immune response. Therefore, different adjuvants namely, Quil A, aluminium hydroxide, or polylactide co glycolide acid (PLGA), were employed. Among all these adjuvants, PLGA was found to be the most effective adjuvant [16]. Further, encapsulation of GnRH-TT in PLGA microspheres induced an effective antibody response within 15 days of administration, thus negating the need of repeated injections [17]. Use of any of the combinations in different animals (rats, monkeys, and pig) was effective in reducing testosterone levels to castration levels thus inducing infertility. However, decrease in testosterone levels was accompanied with concomitant atrophy of prostate.

Therefore, vaccines targeting GnRH were developed primarily as immunocontraceptive and immunocastration agents in animals [18]. Few GnRH vaccines are already available for veterinary use such as Vaxstrate and Improvac whilst research is still under development to obtain potentially improved vaccines [19–21]. In humans, they can be used for the treatment of prostate cancer and various sex hormone-dependent disorders [22]. However, the inability of vaccine to induce azoospermia in all the cases, loss of body hair, atrophy of prostate, and impotency after chronic exposure hinders its application as a safe vaccine. Also, as the vaccine affects sex steroids, consumers are unlikely to find this approach acceptable. Therefore, this may be suggested that enough data needs to be accumulated before the vaccines against GnRH can be employed in humans without side effects.

#### 2.1.2. Follicle Stimulating Hormone (FSH)

Follicle stimulating hormone (FSH) plays an essential role in the initiation and maintenance of spermatogenesis in primates [23]. FSH has two subunits alpha (*α*) and beta (*β*). The *α* subunit of FSH, LH, thyroid stimulating hormone (TSH), and human chorionic gonadotropin (hCG) is identical. FSH-*β* is responsible for its binding with FSH receptor.

The use of FSH appears to be an attractive contraceptive candidate for application in males. Most of the research has been carried out using ovine FSH rather than human FSH. The rationale for this is associated with the fact that specific antibodies raised to ovarian FSH cross reacted and neutralized the activity of FSH of variety of species including humans. Also, the use of ovine FSH in place of human FSH lowers the chances of producing cross reactive antibodies with human LH and TSH (due to structural similarity with *α*-subunit) [24]. Immunization of male bonnet monkeys with ovine FSH resulted in testicular dysfunction, oligozoospermia, and subsequent infertility [25]. It was noteworthy that infertility was not associated with any change in testosterone levels thus adding an additional advantage of using FSH based vaccine. Following successful studies by ovine FSH, a phase 1 clinical trial was carried out using two different versions either ovine FSH *α* *β* heterodimer or isolated ovine *β*-chain. In both the cases alum was used as an adjuvant [26]. Immunization by any of these resulted in generation of FSH-specific antibodies in all immunised individuals. But, as the antibodies being generated were of fairly low titer, there was no reduction in the sperm count in the volunteers.

Further, an attempt was made by using FSH coding peptides instead of native protein form. Two peptides considered contained the sequences of *β* 33–53 and *β* 81–95 aa. Both the peptides were used in monomeric as well as dimeric form (tandem) and were subsequently used to immunize male rabbits, respectively. Immunization with *β* 33–53 tandem inhibited FSH bioactivity specifically and hence it was considered an attractive candidate to be used as the antigen of choice in a male contraceptive vaccine [27]. However, incidence of spermatogenesis in FSH-*β* knockout mice and considerable fertility in men homozygous for an inactivating mutation of FSH receptor [28] suggested that FSH suppression might not be a feasible alternative for the development of male contraceptive vaccine.

#### 2.1.3. Luteinizing Hormone (LH)

Luteinizing hormone (LH, also known as lutropin and sometimes lutrophin) is a heterodimeric glycoprotein hormone produced by gonadotroph cells in the anterior pituitary gland. An acute rise of LH triggers ovulation and development of the corpus luteum in females. It stimulates leydig cells to produce testosterone in males and also termed as interstitial cell-stimulating hormone (ICSH) in males. Similar to FSH, LH is also made up of one *α* and one *β* subunit which make the full, functional protein. Immunization of adult female sheep (ewe) with LH prohibited pregnancy in all the animals during two breeding seasons. The mode of action was assumed as prevention of ovulation presumably by inhibition of the preovulatory surge of LH [29]. Though immunization with LH has potential application as contraceptive agent, it is not widely accepted since it potentially affects sex steroids.

### 2.2. Gamete Outcome

The fusion of sperm and ovum is followed by gamete outcome, that is, the formation of fertilized egg which is maintained under the control of pregnancy specific enzyme, human chorionic gonadotropin (hCG). Soon after the conception, hCG is secreted during luteal phase and prepares endometrium to receive fertilized egg. Generation of antibodies against hCG can provide an immunocontraceptive agent which will target the establishment of fertilized egg.

Human chorionic gonadotropin is the only well-defined protein playing an essential role in the maintenance of pregnancy. The hormone is formed by the trophoblast and acts on the corpus luteum for stimulating progesterone production. If progesterone level drops, uterine endometrium will shed; thus preventing implantation [30]. As the substantial amounts of hCG are secreted only during pregnancy, its neutralization should have no other effects than on fertility. It also plays a critical role in implantation of the embryo onto the uterus.

Anti-hCG vaccine was the first and only birth control vaccine to go through Phase II efficacy trials successfully. The use of vaccine in sexually active women prevented them from becoming pregnant [31]. The vaccine was highly effective as long as the antibody titers remained >50 ng/mL. Action of the vaccine was fully reversible as women conceived readily when the antibody titers declined (<35 ng/mL). No side effects were observed during Phase I and Phase II trials as women showed normal ovulation and normal synthesis of their own sex hormones along with regular menstrual cycles. Also, no irregularity of bleeding in terms of spotting, amenorrhoea, or extra bleeding occurred [32].

Despite the encouraging results of phase I and phase II trials, major shortcoming of vaccine still existed which included the variation in generation of protective threshold titers. Only 60–80% women showed the presence of high titer protective antibodies. This percentage would have been satisfactory in case of infectious diseases but in case of a birth control vaccine, it has to be effective in >90–95% of recipients in order to be acceptable.

Therefore, in order to enhance the titers, recombinant approaches were tried using different carriers. Use of both diphtheria toxin (DT) and tetanus toxin (TT) evokes good antibody response but the repeated immunization with *β*-hCG-TT vaccine causes a carrier induced epitope suppression in some women. Various studies showed that conjugation of *β*-hCG to cocktail of peptides resulted in enhanced immune response as compared to single peptide [33].

Later, another study was carried out using heat labile enterotoxin subunit B (LTB) genetically linked with hCG-*β* chain. It was cloned and expressed in yeast* Pichia pastoris* and was purified using standard purification techniques [34]. Intramuscular immunization in Balb/c mice fortnightly resulted in generation of bioeffective anti-hCG antibodies. The titers when tested were several folds higher than 50 ng/mL/mouse. Similar results were obtained when this vaccine was used in different inbred mice of varying genetic background. The action of the vaccine was reversible and it received approval from the Indian National Review committee on Genetic Manipulation. The vaccine is being produced under GMP conditions for the preclinical toxicological studies. After passing these tests, next plan is to conduct clinical trials with the vaccine for preventing pregnancy, as well as for its possible therapeutic action on cancers expressing hCG or its subunits [35].

### 2.3. Gamete Function

The term gamete function is associated with the two main cells participating in gamete formation, that is, sperm and ovum. This approach targets the zona antigens of egg and surface antigens of sperm. The targets being nonhormonal provide much better safety upon other approaches. Exploiting ZP antigens as immunocontraceptive may interfere with the normal production of egg in female while sperm-based vaccine will be used in female reproductive tract to block fertilization by interfering with sperm movement or function.

#### 2.3.1. Zona Pellucida (ZP)

ZP is an extra cellular matrix, composed of glycoproteins, which surround the mammalian oocyte. This matrix serves multiple functions which play important role during fertilization. It is responsible for binding of spermatozoa to the oocyte due to presence of specific glycoproteins on its surface which acts as a receptor for spermatozoa. This interaction is followed by induction of acrosome reaction in the sperm bound to ZP which results in successful fusion between the two. ZP is composed of 3-4 glycoproteins named ZP1, ZP2, ZP3, and ZP4 [36].

**Figure 2:**
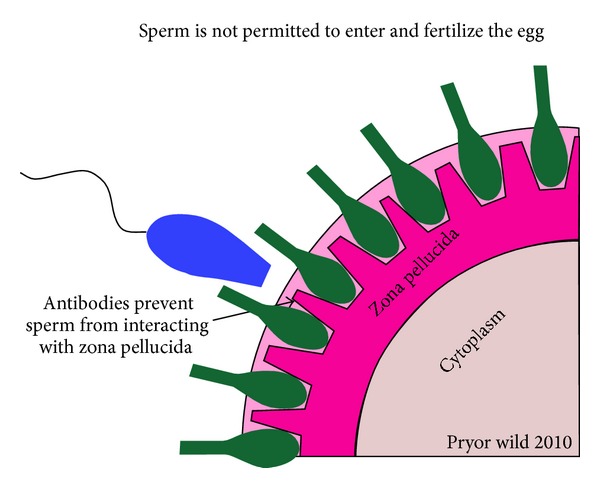
Immunocontraception using an immune response to zona pellucida (ZP). The immune response to the ZP antigen can inhibit sperm-egg binding or disrupt ovarian function (Source: http://www.newmalecontraception.org/risug/).

The ZP glycoproteins have emerged as potential candidate for immunocontraception due to their essential role in the fertilization and tissue specificity (Figure 2). Immunization against ZP might result in generation of antibodies against themselves which go and bind to ZP. Thus, when a sperm encounters ovum in case of immunized animals, the binding is inhibited because ZP is already occupied by the antibodies. Immunization of female rabbits with porcine ZP proteins results in generation of antibodies against porcine ZP proteins which showed immunological cross reactivity with ZP from various other species including humans. Further, immunized female rabbits failed to conceive even after mating with fertile male rabbits [37]. This homology allowed the use of heterologous immunization in case of ZP [7].

Further studies revealed that the infertility induced in immunized female rabbits was irreversible which could not be restored even after the administration of exogenous gonadotropins. Histological examination of ovaries showed the destruction of oocytes in all the growing follicles along with the depletion of resting follicles [38]. This observation indicated that the infertility was a consequence of ovarian dystrophy rather than inhibition of sperm-oocyte interaction.

The irreversibility associated with immunization using ZP posed a major hurdle in the development of ZP based contraceptive. While irreversibility is not a major concern in case of wildlife management where long term infertility is often desirable. Therefore further development in this area resulted in production of various marketed products playing an imperative role in wildlife management.

The immunization of female bonnet monkeys was carried out using purified porcine ZP3. High antiporcine ZP3 antibody titers were formed and all the animals were rendered infertile [39]. Only 50% of the animals could regain fertility after the decline in antibody titers.

Ovarian histology of the animals that failed to regain fertility did not reveal any signs of inflammation or lymphocytic infiltration. The observed variations in the extent of ovarian dysfunction may be linked with differences in susceptibility among various species, purity of the ZP glycoproteins, and use of different adjuvants such as alum and synthetic muramyl dipeptide (MDP) [40–42]. It was suggested that these problems such as limited production of ZP glycoproteins from native source, batch to batch variation, and contamination with ovarian-associated proteins might be solved using recombinant form of ZP.

For this, the three glycoproteins, namely, ZP1, ZP2, and ZP3, were separately expressed in Chinese hamster ovarian (CHO) cells. A comparative analysis was carried out in two nonhuman primate species, namely, cynomolgus monkeys (*Macaca fascicularis*) and baboons (*Papio cynocephalus*) [43]. Animals immunized with any of the recombinant proteins remained infertile for some period of time but the animals immunized with recombinant ZP1 conceived later in contrast to control animals and animals immunized with remaining two proteins. Hence, another study was carried out in female baboons (*Papio anubis*) and immunization was done using* E. coli*-expressed recombinant bonnet monkey (*Macaca radiata*) ZP1 (bmZP1) conjugated to diphtheria toxoid (DT) [44]. After mating with males of proven fertility, immunized animals failed to conceive till the antibody titers were >2 × 10^3^ antibody units. All the immunized animals became pregnant upon mating once the levels were less than the 2 × 10^3^ antibody units. Significant curtailment of fertility was also observed by using recombinant possum ZP3 in grey kangaroos [45, 46]. Though the results were quite exciting, histological examination of ovaries of immunized animals revealed the presence of atretic follicles with degenerating oocytes.

These observations raised the hope of generation of ZP based CV for human use except for the histopathological damage. Thus, series of experiments were conducted to overcome these problems. It was found out that “oophoritogenic” T-cell epitopes existing in zona proteins might be responsible for ovarian dysfunction [47]. Hence, efforts were made to delineate B-cell epitopes which were devoid of “oophoritogenic” T-cell epitopes. As a result various synthetic peptides corresponding to ZP glycoproteins (ZP1, ZP2, and ZP3) were prepared and tested for immunocontraceptive potential* in vivo*. Female bonnet monkeys immunized with synthetic peptides remained infertile. Histopathological examination showed absence of any ovarian pathology in case of immunized animals. Similar results were obtained in case of mice immunized with ZP3 [48, 49].

Recently, murine ZP3 and sperm specific (YLP-12) epitope were expressed with Johnson grass mosaic virus coat protein to present antigens as virus like particles (VLPs) [50]. Immunization of animals resulted in significant infertility. The results were quite encouraging and suggestive of the possibility of using above mentioned approaches for immunocontraception. However, long term studies showed that immunization with zona antigens might induce immunological attack on many eggs in the ovary which might lead to premature ovarian failure [51]. Hence, enough data needs to be gathered before zona antigens are given any place in the market as immunocontraceptive measure [52].

#### 2.3.2. Sperm Antigens

Development of CV based on sperm is a promising approach towards contraception [53]. The feasibility of employing sperm as a target is well documented. Sperm has both auto- and isoantigens and hence immunization of several species of animals and humans with sperm results in generation of antisperm antibodies (ASA), leading to infertility [54]. In a classic study carried out in 1932, fertile women (had at least one pregnancy) were injected with their husbands semen [55]. The women developed ASA which resulted in block of conception for up to 1 year of observation. More than 70% of the vasectomised men produce ASA after vasectomy which interferes with regain of fertility even after successful surgical reanastomosis [56]. In addition, approximately 30% infertility may be attributed to the presence of ASA in either male or female partner. Disappearance of ASA from these males and females results in the recuperation of fertility. These findings provide evidence that sperm is an attractive candidate that can be exploited in case of both men and women.

Exploitation of whole spermatozoon/poorly characterized/crude sperm extracts is not a practical and feasible approach for the CV development as it has several antigens that are likely to be shared with various somatic cells due to molecular mimicry [6, 57]. Immunization with whole sperm can lead to immunopathological consequences with other tissues and organs. Thus, only sperm specific antigens can be regarded as a promising and attractive candidate for CV. The utility of an antigen is contingent upon its immunogenicity, expression on sperm surface (except from the acrosomal antigens that appear after the acrosome reaction) and involvement in fertility/fertilization. Fertilizing capacity of spermatozoa is dependent upon wide range of biological properties, namely, motility, ability to capacitate, undergoing acrosome reaction, penetrating ZP, and finally fusion with egg [58]. Antibodies generated against any of the antigens involved in above said phenomenon can result in suitable candidate.

Various approaches of genomics, proteomics, and vaccinology are being used to isolate sperm-specific antigens. In addition, several novel technologies such as subtractive hybridization, hybridoma technology, differential display technique, and gene knockout technology have also been employed. In addition to these approaches, sperm-bacteria interactions can also be targeted for the delineation of sperm antigens. Among various microorganisms that directly interact with spermatozoa leading to sperm immobilization/agglutination are well known causative pathogens of genitourinary infections such as* Chlamydia trachomatis*,* Mycoplasma genitalium*,* Mycoplasma hominis*,* Ureaplasma urealyticum*,* Pseudomonas aeruginosa*,* Neisseria gonorrhoeae*,* Escherichia coli*, and* Staphylococcus aureus* [59]. These interactions use specific host cell-surface molecules known as receptors and such receptors provide a mechanism for intimate interaction with the ligand on bacteria. Harvey et al. [60] while studying the mechanism of interaction in case of* N. gonorrhoeae* with sperm showed that* N. gonorrhoeae* lipooligosaccharide (ligand) can bind to the asialoglycoprotein receptor (ASGP-R) on human sperm. Sulphoglycolipids have been confirmed as the only receptor molecule for the 70 kDa heat shock protein ligand on* M. hominis* [61]. Earlier studies have shown that sperm-*E. coli* adherence is mediated by mannose residues present on the sperm surface and mannose binding structures present on* E. coli* [62]. Addition of receptor antagonist resulted in inhibition of sperm-bacteria interactions. Therefore, it can be concluded that interaction of bacterium with spermatozoa is receptor mediated and involves interplay of multiple factors which cumulatively lead to sperm damage.

Amongst these microorganisms,* E. coli* is perhaps the most extensively studied microorganism in relation to infertility [63].* E. coli* rapidly adheres to and agglutinates human sperm which results in significant decrease of motile sperm count. On the basis of this chemistry involved, the possibility arises that bacteria can also be exploited to identify these proteins on spermatozoa.

Various antigens that are being isolated from spermatozoa include sperm adhesion molecule 1 (SPAM 1), metalloprotease disintegrin cysteine (MDC), sperm protein (SP-10), fertilization antigen (FA-1), SP-17, NZ-1, NZ-2, lactate dehydrogenase (LDH-C_4_), sperm agglutination antigen (SAGA-1), YLP-12 peptide, human equatorial segment protein (hESP), BS-17, rabbit sperm membrane protein-B (rSMP-B), sperm acrosomal membrane-associated protein (SAMP-32), and 80 kDa human sperm antigen (HSA). In addition few glycoproteins on the surface of spermatozoa were acquired from epididymal secretions during transit through epididymis, namely, dorsal head and equatorial (DE), epididymal protease inhibitor (Eppin), and sperm flagella protein (SFP-2). Izumo and AKAP are also being isolated.

Following antigens are being tried as CV. The antigens so obtained were tried in purified/semipurified forms for immunization. However, to achieve Food and Drug Administration (FDA) approval and to perform appropriate multicenter fecundity trials in a quality-controlled manner, recombinant or synthetic peptide molecules are required. Hence in addition to the use of native form, alternative forms of protein/antigens, namely, recombinant proteins and synthetic peptides, have also been tried. Among these two forms, synthetic form possesses advantage of being well-defined which can be synthesized and purified in large quantities at a relatively lower cost in contrast to recombinant proteins.

*(1) The Sperm Adhesion Molecule 1 (SPAM1)*. The sperm adhesion molecule 1, a 64 kDa glycosyl-phosphatidylinositol (GPI)-linked protein, is expressed in testis, epididymis, sperm, and luminal fluid of the epididymis [64, 65]. SPAM1, a hyaluronidase enzyme, carries out three main functions during fertilization. These include penetration of cumulus [66], binding with ZP, and Ca^2+^ signalling in acrosomal exocytosis [67]. The protein is widely conserved among various mammals, hence it has been cloned from many species including fox [68], guinea pig [69], pig [70], rat [71], rabbit [72], monkey, and man [73].

*In vitro* incubation of sperm with antibodies generated against SPAM1 resulted in reduced sperm-ZP binding. Immunization of either male or female guinea pigs with purified SPAM1 from guinea pigs induced 100% infertility [74]. Infertility was associated with loss of normal sperm in epididymis and autoimmune disorders in case of immunized males [75]. In females, prevention of sperm-egg binding by antibodies resulted in infertility. However, the infertility induced by immunization was found to be reversible in both the cases as fertility was regained within 6–15 months.

Though the results were quite encouraging, but in order to produce an effective CV, it is necessary to generate protein in sufficient quantities. This shortcoming has been undertaken by the use of recombinant antigen. The cDNA encoding for the SPAM1 antigen has been cloned and sequenced. However immunization of both male and female rabbits carried out using recombinant SPAM 1 (rSPAM-1) did not result in infertility in any of the immunized animal. These differences between native and recombinant form may be due to lack of sufficient quantities of anti-rSPAM1 antibodies which were required to block conception [72, 76].

*(2) Metalloprotease/Disintegrin/Cysteine-Rich (MDC)*. The MDC proteins are a rapidly growing family of integral membrane proteins, expressed predominantly in mammalian testis. All the proteins have distinct conserved features such as a metalloproteinase-like domain, a disintegrin-like domain, a cysteine-rich domain, a prodomain, and a transmembrane domain (also known as ADAM family) [77]. Among these proteins, few are known to be expressed on male germ cells and/or mature sperm. Notable among them are fertilin *α*, fertilin *β* (collectively called fertilin), and cyritestin (tMDC I).

Fertilin, previously designated PH30, is a sperm-associated protein. It was originally isolated from detergent extracts of guinea pig caudal epididymal sperm. In guinea pig, it is composed of two related subunits, *α* and *β*, with molecular masses of 60 kDa (Fertilin *α*) and 44 kDa (Fertilin *β*), respectively, as observed on reducing sodium dodecyl sulphate-polyacrylamide gel electrophoresis (SDS PAGE). Role of fertilin *α* is associated with sperm-egg adhesion, but whether it has some part to play in membrane fusion or not is still not very clear [78]. Its role in adhesion is justified by the presence of cysteine-rich domain that appears to participate in cell adhesion. The use of recombinant form of fertilin *α* inhibits sperm-egg binding more effectively than does a shorter form with a truncated disintegrin-like domain. But the fact that the sperm lacking fertilin *α* are still capable of sperm-egg fusion has hindered the use of fertilin *α* [79].

Fertilin *β* was one of the first “cellular disintegrins” identified. It is one of the subunits of dimeric sperm antigen “fertilin” and the antibodies generated against fertilin cross reacts with fertilin *α*. The function of fertilin *β* is well known; it mediates sperm-egg binding and membrane fusion [80]. Inhibition in the presence of antidisintegrin loop antibodies pointed that fertilin *β* appears to utilize its disintegrin loop sequence to interact with the egg membrane. Although antibodies to fertilin *β* inhibit fertilization* in vitro* [81], but immunization with the same does not affect fertility* in vivo*, it is not suitable as a contraceptive agent [82, 83].

Cyritestin is the product of* Cyrn* gene and has an apparent molecular weight of 110 kDa but is subjected to processing during epididymal sperm transport, thus having an approximate weight of 55 kDa. It is involved in sperm-oocyte membrane adhesion and is similar to fertilin; it also uses its disintegrin loop for adhesion. Cyritestin gene knockout mice did not show any defect in sperm-oocyte membrane fusion, but they did show impairment in sperm-zona binding [84]. However, as human cyritestin gene is nonfunctional illustrating the risk of using rodents as models of human/primate fertility.

*(3) Sperm Protein-10 (SP-10)*. Sperm-specific acrosomal protein, SP-10, was first identified in humans [85]. It was observed that SP-10 is a testis but not species-specific protein as antibody against SP-10 recognized sperm from baboon, macaque, and pig, but not from rabbit, bull, rat, guinea-pig, and cat [86]. The cDNA encoding SP-10 protein has been cloned and sequenced from a human cDNA expression library and was found to contain 1117 bp sequence encoding for 256 aa [87].* In vitro* incubation of anti-SP-10 antibodies inhibited bovine fertilisation by reducing sperm-zona binding.

*In vivo* potential of SP-10 as an immunogen was tested using attenuated strain of* Salmonella* sp. expressing human SP-10. Immunization was carried out using oral route in mice and intramuscular route in monkeys. Presence of anti-SP-10 specific antibodies was seen in both cases [88]. However, antibody levels in the oviduct are of particular relevance in case of SP-10 since it is localised within the acrosomal compartment and the outer acrosomal membrane complex and is therefore only accessible to antibody after the acrosome reaction has been initiated.

*(4) Fertilization Antigen-1 (FA-1)*. Fertilization antigen-1 (FA-1) is a 47 kDa glycoprotein which develops in testis during later stages of spermatogenesis and has autophosphorylating activity [89]. This tyrosine phosphorylation occurs during human sperm capacitation/acrosome reaction [90]. Thus, it was suggested that FA-1 has important role in capacitation/acrosome reaction which might also have effect on sperm-ZP binding. To check the specificity of FA-1 towards ZP,* in vitro* incubation of FA-1 antigen was performed with major components of ZP, that is, ZP1, ZP2, or ZP3. Results showed the specificity of FA-1 antigen towards ZP3, thus suggesting the presence of complementary sequence of FA-1 antigen on the murine oocyte.

Further, monoclonal antibodies raised against FA-1 antigen were used to ascertain the phenomenon involved in reduction of fertilization [91]. It was observed that antibodies to FA-1 block fertilization by affecting both capacitation/acrosome reactions as well as reducing sperm-zona binding. This blockage was not limited to humans as similar results were obtained in case of mice, cattle, and monkeys.

Active immunization of mice with rFA-1 resulted in an overall 64–70% reduction in fertility compared to that of controls [92]. The effect induced by antibodies was reversible and long lasting as animals delivered healthy babies when the titers reached control levels with no effect on litter size. Thus, involvement of rFA-1 antigen makes it attractive candidate for CV development. However, further studies in higher animal models are still awaited.

*(5) Sperm Protein 17 (SP-17)*. Three sperm specific low molecular weight (~13 ± 2 kDa) proteins have been isolated from rabbit sperm and named rabbit sperm antigen-1 (RSA-1), RSA-2, and RSA-3/SP17 [93]. Monoclonal antibodies to RSA antigens showed cross reaction with human, baboon, and mice sperm. These antibodies also inhibited penetration of zona-free hamster oocyte by human spermatozoa [94]. Among these, SP-17 is the most important antigen which is exposed on the surface of mouse sperm after acrosome reaction. The localization of SP-17 was seen throughout the equatorial segment and this segment binds to ZP.* In vitro* studies revealed the specificity of SP-17 towards ZP3. When male and female mice were immunized with a chimeric peptide containing SP-17 and T-cell epitope, there was rise in antibody titers. This rise in antibody titer confirmed the immunogenic nature of the peptide. Interestingly, the increase was pertinent in both sexes.

*(6) NZ-1*. The isolation of NZ-1 was carried out using polyclonal sera which was obtained from mice after immunization with human sperm antigens. The antigens so obtained had bands belonging to the molecular weight of 14–18 kDa on SDS-PAGE. Screening of the mouse *λ*gt11 library using these antibodies recognized a cDNA clone coding for an antigen, designated as NZ-1. Neither the sequence of amino acid nor cDNA had matching with any known cDNA/amino acid sequence present in database. Recombinant form of NZ-1 antigen was used to immunize mice which resulted in reduced fertility rates [95].

*(7) NZ-2*. Another cDNA encoding for a sperm antigen, designated NZ-2, was also cloned and sequenced from human testis cDNA-lgt11 expression library by using polyclonal antibodies to human sperm surface antigens belonging to 14–18 kDa molecular regions. These sperm antigens are involved in binding to ZP of the human oocyte. The NZ-2 cDNA has 335-bp 58 and 139-bp 38 noncoding regions. The translated protein has a calculated molecular weight of 19 kDa. Extensive computer search in the Gen-Bank, National Biomedical Research Foundation (NBRF), and Swiss database indicates it to be a novel protein, having 99.5% nt sequence similarity, except for the first 40-bp, only with the human bacterial artificial chromosome (BAC) containing cloned human sperm DNA. The 20 kDa protein was recognized specifically by the antisperm IgG, not by the control IgG in the western blot procedure [92]. The recombinant human sperm NZ-2 antigen may find applications in the development of a contraceptive vaccine and diagnosis and treatment of infertility in humans.

*(8) Lactate Dehydrogenase (LDH-C*_*4*_ *).* The isozyme lactate dehydrogenase (LDH) plays important role in lactate metabolism and glycolysis of developing and mature spermatozoa. It has different subunits LDH-A, LDH-B, and LDH-C. Among these, LDH-C subunit is independent gene product which is expressed only in spermatogenic cells and is immunologically different from the other two subunits. Homotetrameric LDH-C_4_ is perhaps the most extensively characterized sperm antigen which plays chief role in lactate metabolism and glycolysis.* In vitro* incubation of anti-LDH-C_4_ antibodies resulted in sperm agglutination and cytotoxic effects on sperm cells. Active immunization using LDH-C_4_ in variety of mammalian species including primates did affect fertility. Immunization of fertile baboons with synthetic form of LDH-C_4_ conjugated to DT resulted in 75% reduction in fertility rates [96].

*(9) Sperm Agglutination Antigen-1 (SAGA-1)*. Sperm agglutination antigen-1 was isolated from human spermatozoa using a monoclonal antibody generated against human sperm, designated as S19. Molecular weight of SAGA-1 ranges between 15 and 25 kDa. Immunolocalization by electron microscopy revealed the reactivity of S19 monoclonal antibody to the entire human sperm surface indicating the presence of SAGA-1 on whole sperm surface [97].* In vitro* incubation of antibody generated against SAGA-1 resulted in agglutination/immobilization of sperm as well as inhibition of penetration of zona-free hamster ova [98].

*(10) Human Equatorial Segment Protein (hESP)*. Human equatorial segment protein (hESP) is a sperm specific protein, first reported in the year 2003 [99]. Mouse ESP (mESP) has 81% homology with hESP. It is localized to the equatorial segment of acrosome and is associated with acrosome biogenesis. Interaction of hESP with sera of infertile male and female patients showed positive reaction. The use of antiserum raised against recombinant hESP inhibited the binding and fusion of human sperm in the hamster egg penetration assay [100]. These results suggested that ESP might be an interesting target for designing contraception vaccine.

When immunization of mice was carried out with polyepitope antigens, results showed that fertility of mice was significantly reduced. Further, an attempt was made to isolate the important part of mESP playing vital role in infertility. For achieving this, full mESP was divided in 3 fragments, namely, P1, P2, and P3. Inhibition of sperm-egg binding was seen only in the presence of anti-P1 or anti-P2 antibodies, while no inhibition was seen in case of anti-P3 antibodies. These results indicated that additional studies are needed to better characterize these peptides [100].

*(11) YLP-12*. A dodecamer sequence (YLPVGGLRRIGG), designated as YLP-12, was identified on human sperm using phage display technology [101]. This technology is an innovative tool which was first reported by George Smith in which the peptide sequences are presented on the surface of filamentous phage to examine their interaction with specific antibodies [102]. The peptide sequence of YLP-12 is primarily localized on acrosome and tail in case of humans and murine sperm. Extensive computer search in the database did not result in homology or identity with any known sequence indicating the novelty of the peptide. Immunoblotting analysis suggested that antibodies resulting from vaccination with YLP-12 recognized a specific protein band of 72 kDa in testis.

*In vitro* incubation of spermatozoa with YLP-12 Fab significantly reduced acrosome reaction (AR) [101]. Prevalence of antibodies against YLP-12 in sera of immunoinfertile patients highlighted its potential to be used as CV. Thus* in vivo* studies were carried out in mouse model using synthetic YLP-12 peptide conjugated with the binding domain of recombinant cholera toxin subunit B (rCTB). Immunization of female mice was done using two different routes, namely, intranasal and intramuscular. Both the routes resulted in overall reduction of 70.3% (intranasal) and 61.4% (intramuscular) in fertility, respectively. Though, further studies need to be carried out in higher animal models.

*(12) Rabbit Sperm Membrane Protein-B (rSMP-B)*. One of the components with a molecular weight of 20.1 kDa was identified from the tail of rabbit sperm and designated as rSMP-B [103]. Immunolocalization of rSMP-B on spermatozoa and somatic cells was tested using polyclonal antibodies to rSMP-B. The results suggested that this antigen was produced by germ cells during spermatogenesis. Monoclonal antibodies raised against rSMP-B were demonstrated for their ability to block the penetration and fertilization of zona-free hamster eggs by human sperm* in vitro*. Immunization of male rabbits with rSMP-B protein resulted in blockage of spermatogenesis resulting in azoospermia. Further it was found that immunization by rSMP-B 230 peptide resulted in 83.3% infertility which was observed in case of immunized female rats after mating.

*(13) Sperm Acrosomal Membrane-Associated Protein (SAMP-32)*. A novel human sperm membrane antigen, SAMP32 (sperm acrosomal membrane-associated protein 32), was discovered from human sperm extracts [104]. When the experiments were carried out to reveal the expression of SAMP-32, results showed that the expression was testis-specific and subsequent stages of acrosome biogenesis. Before acrosome formation, SAMP32 expression did not occur.* In vitro* incubation of anti-SAMP antibodies significantly suppressed the binding and the fusion of capacitated human spermatozoa with zona-free hamster eggs in comparison to preimmune serum. The reason that serum from an ASA positive infertile man strongly reacted with the recombinant SAMP32 antigen suggested that it might be one of the antigens related to immune infertility which can be exploited as a target for CV [105].

*(14) 80 kDa Human Sperm Antigen (80 kDa HSA)*. 80 kDa HSA was identified from human sperm extract by western blot analysis using serum of an immunoinfertile healthy woman. It was found to be a conserved glycoprotein localized only in testis and epididymis and not localized in other somatic tissues. The pI of the protein was found to be 4.5 [106]. Active immunization of both male and female rats was carried out using native protein and it induced infertility in both sexes. Use of synthetic form of 80 kDa HSA was also tried in order to augment the immunogenicity. Maximum inhibition in fertility was seen in case of male rabbits immunized with peptide-1 followed by peptide-NT and remaining peptides. Active immunization of male marmosets with synthetic peptide-1 induced antibody response in 7 out of 9 marmosets and 6 out of 7 became infertile. Semen samples of the animals from treated group showed complete loss of progressive motility. The effect was found to be reversible as antibody titer declined 8–10 weeks after last booster injection and the animals regained their fertility. Thereafter, no apparent effect on normal physiological processes was observed [107].

*(15) BS-17*. BS-17 is one of the antigens being identified using ASA from serum of infertile woman. When the human testis *λ*gt11 cDNA expression library was probed with polyclonal anti-BS-17 antibodies, the positive clone yielded a cDNA fragment consisting of 758 bp. Polyclonal antibodies to the BS-17 antigen inhibited human sperm from penetrating and fertilizing zona-free hamster oocytes [108]. Due to 99.7% homology of BS-17 with calpastatin, it was suggested that this inhibition involved destabilization of the calpastatin-calpain complex by the anti-BS-17 antibodies. After this destabilization, calpain could trigger the premature sperm acrosome reaction before sperm would reach the ovum, thus resulting in deterioration of the sperm fertilizing capability. However, till now none of the* in vivo* experiments have been carried out.

*(16) Contraceptive Vaccinogen*. Complementary DNAs encoding few sperm antigens have been cloned and sequenced [92]. Subtractive cDNA hybridization technology was used to isolate novel human testis specific antigen which was designated as contraceptive vaccinogen [109]. The molecular mass of the deduced aa sequence of contraceptive vaccinogen protein was calculated to be 35.3 kDa. Contraceptive vaccinogen cDNA had no homology with existing sequences in database.

The recombinant contraceptive vaccinogen protein recognized ZP3 component of ZP of human oocytes rather than ZP1/ZP2. The specificity of this interaction was further confirmed when human sperm binding to ZP of human oocytes was significantly inhibited in the presence of antirecombinant contraceptive vaccinogen antibodies. In addition, immunobead binding technique (IBT), using live sperm, highlighted the predominant presence of recombinant contraceptive vaccinogen on surface, thus accessible to antibody binding. Though antibodies produced against recombinant contraceptive vaccinogen did neither agglutinate nor cause immobilization, they could block capacitation of human sperm as deduced by sperm penetration assay (SPA). Presence of contraceptive vaccinogen antigen on murine sperm raises the feasibility of exploitation of animal model for studying its immunocontraceptive potential and it may find application in CV development.

*(17) Epididymal Protein-20 (EP-20).* A 20 kDa glycoprotein designated as epididymal protein (EP-20) was isolated from rabbit cauda epididymal fluid. Immunolocalization of EP-20 protein was carried out using polyclonal antibodies to EP-20 which showed the presence of the protein in testis and epididymis. Interestingly, intense staining was also observed in case of human testes, whereas germ cells, interstitial cells, and nine other tissues remained unstained. This observation highlighted the conserved nature of the protein. Polyclonal antibodies raised against EP-20 not only immobilized and agglutinated human sperm but also blocked the penetration of zona-free hamster eggs by human sperm* in vitro* [110]. However, potential of EP-20 as an immunocontraceptive needs to be examined in* in vivo* model.

*(18) Dorsal Head and Equatorial Segment (DE) Protein*. Dorsal head and equatorial segment protein, also known as acidic epididymal glycoprotein (AEG), has a molecular weight of 37 kDa. It is associated with the dorsal region of the sperm head during epididymal maturation with binding characteristic of a receptor-type mechanism [111]. During acrosome reaction both* in vitro* and* in vivo*, the protein migrates from epididymal region to the equatorial segment of sperm [112]. The protein plays an important role in sperm-egg binding. Incubation of sperm with antibodies against the protein significantly inhibited the penetration of zona-free eggs* in vitro* [113]. This binding was significantly reduced when sperm formerly exposed to anti-DE antibodies were used for artificial insemination* in vivo* [111]. Decreased fertilization rates were observed in male rats, after immunization with purified DE.

Further an attempt was made to evaluate the immunocontraceptive potential of recombinant DE in rats. Animals immunized with recombinant form showed statistically significant reduction in fertility in contrast to native DE. This observation supports the evidence of exploiting epididymal proteins as a target for CV development.

*(19) Epididymal Protease Inhibitor (Eppin)*. An epididymal protein designated as epididymal protease inhibitor (Eppin) has a molecular weight of 26 kDa [114]. Immunizationof male monkeys was carried out using Eppin. After immunization, 78% of monkeys who developed high anti-Eppin antibody titers became infertile and 71% of those monkeys recovered fertility after immunization was stopped. Later, another study was carried out wherein active immunization with Eppin gave maximum reduction of 90% in animals [115]. These results highlighted the potential of Eppin as a very attractive candidate in contraception because none of the proteins isolated so far resulted in >75% of infertility. To enhance the sensitivity of eppin, recombinant form of Eppin was tried. However, immunization with recombinant protein resulted in only 70% of reduction in contrast to native protein. Therefore, further studies need to be employed using Eppin as a potential target for immunocontraception.

*(20) Sperm Flagella Protein (SFP2)*. Several novel epididymis-specific proteins have been identified using the combinatorial approach [116]. SFP2 is localized on the surface of sperm and showed epididymis specificity. Antibodies to SFP2 peptide 1 recognized a doublet at 220–230 kDa only in epididymal extract. Antibody to peptide 1 recognized the cognate protein on mouse, rat, and human spermatozoa. Immunization of male mice with peptide1 resulted in production of specific antibodies.

*In vitro* incubation of immune sera from immunized male mice with sperm caused significant reduction in motility and viability but did not agglutinate sperm. Mating of immunized male mice was allowed with female mice. A reduced fertility rate of 20% was observed in female mice mated with immunized male when compared with control animals (100%). The antibody levels in the immunized males declined by 22 weeks after immunization, resulting in 100% reinstatement of fertility. Histological examination of reproductive organs of the immunized mice and control mice caput, corpus, and cauda region of epididymis was carried out. No gross histological difference was found between immunized group and control group. No sign of leucocyte infiltration was seen in any tissue thereby; data provide an experimental basis for the development of an effective contraceptive vaccine based on new epididymal target. Still, the research is at its infancy stage and has miles to go.

*(21) Izumo*.* Izumo* is named after a Japanese shrine dedicated to marriage. It is a typical type I membrane glycoprotein consisting of one immunoglobulin, like domain and putative N-glycoside link motif. It is expressed on both mouse and human sperm with a molecular weight of 56.4 kDa and 37.2 kDa, respectively. cDNA encoding for mouse and human Izumo protein has been sequenced. It is not detectable on ejaculated sperm but becomes recognizable once the sperm cell undergoes AR. Among all gene knockouts, Izumo has gained highest insight because gene knockout male mice for Izumo resulted in 100% block of conception. It was observed that even after mating of Izumo−/− male mice with proven females, none of the female delivered pups [117].

Thus to evaluate the immunocontraceptive potential of Izumo, three peptides of Izumo, namely, IZ1/IZ2/IZ3, were generated [118]. High titer specific antibodies were generated against all the peptides in three groups in contrast to control group (nonimmunized). Mating of immunized female mice with proven breeder male mice was allowed, and results showed block of conception leading to infertility. However, none of the peptides resulted in 100% contraceptive effect and maximum contraceptive effect was found to be only 53.4%. Regain of fertility was seen few months subsequent to the last booster. Thus, it may be stated that Izumo antigen holds potential for becoming CV. Also, as it is not exposed until the sperm cell undergoes AR; hence antibodies against the protein should be present at a particular space and time.

*(22) A-kinase Anchoring Protein (AKAP)*. Phosphorylation of sperm proteins is regulated by a synchronized balance between kinase and phosphatase activities. It was demonstrated that two proteins, namely, A-kinase anchoring protein 3 (AKAP 3) and AKAP 4, are the most abundant proteins present in fibrous sheath of sperm. AKAP 3 is the basic organized structure of the sheath while AKAP 4 plays an imperative role in completing the assembly of fibrous sheath [119]. Relation of partial deletions of Akap3 and Akap4 gene sequences may therefore be linked to defective assembly and failure of compartmentalization of AKAP3 and AKAP4 proteins in the tail. These gene deletions may result in sperm immotility [119, 120]. However, the hypothesis needs to be verified using animal models.

*(23) Testis Specific Antigen-1 (TSA-1)*. A 24 kDa testis specific antigen-1 (TSA-1) is expressed in murine sperm and human sperm. Northern blot procedure revealed the expression of TSA-1 on testis. TSA-1 cDNA did not have any sequence similarity with known nucleotide/amino acid sequence in the databases. Antibody recognized acrosomal, equatorial, mid piece, and tail regions of human sperm [121]. Antirecombinant TSA-1 antibodies inhibited the acrosome reaction and sperm egg binding in* in vitro* assays [122]. These findings indicated that the testis/sperm specific protein has role in human sperm function and may find clinical application in the contraceptive vaccine development.

Various sperm antigens have been delineated and characterized. Active immunization using most of these antigens resulted in generation of high titer specific antibodies; even then none of the antigens have resulted in 100% infertility. The reasons associated include variability in immune response at individual level, maintenance of high antibody titers, time lag to achieve reasonably good titers, that is, approximately 3 months, and uncertainty that for how long the required level of antibody will remain in circulation. All these concerns may be taken care by the use of preformed antibodies, that is, passive immunization approach. The success of application of passive immunization in case of various infectious diseases indicated the feasibility and viability of such an approach [123]. Furthermore, local application of preformed antibodies will not interfere with endocrine system of the individual and is independent of host's immune status, thus providing additional advantages over the use of active immunization.

### 2.4. Passive Immunization: An Effective Approach

Passive immunization includes two basic approaches, that is, use of polyclonal or monoclonal antibodies. In comparison to polyclonal antibody, use of monoclonal antibody is particularly appealing and feasible. Being specific, monoclonal will avoid any cross reaction with somatic cells and there will also be lesser chances of allergic reactions. Due to this reason, they have been heralded as “magic bullets.” The term “magic bullet” was coined in the end of 19th century to describe a theoretical agent that could specifically target pathogens while leaving a healthy tissue intact [124]. As a further consequence of their high specificity, monoclonal antibodies are more potent; for example, use of monoclonal (Synagsis) against RSV infection in high risk infants is approximately 50 times effective than polyclonal preparation (RespiGam). Similar results were obtained in case of* Streptococcus* infection in neonates. Also, recent advances in hybridoma technology used for generation of monoclonal antibody have revitalized interest in passive immunization.

The guiding concept which led to the idea of using passive immunization for immunocontraception was based on the clinical reports of infertile men whose semen contains antibodies that immobilize sperm usually by agglutination. The data from previous studies indicated that sperm agglutinating/immobilizing antibodies must be present in the reproductive tract specifically vagina to provide significant immunoprotective effect from pregnancy. Earlier reports have examined the stability of antibodies in mucus secretions and found that antibodies whether polyclonal/monoclonal are stable in seminal fluid and cervical mucus at 37°C. The antibodies can provide protection for one to several days depending on their half-life [125] and the use of gels for the delivery of antibodies will result in sustained release.

Very few antigens have been explored to date via passive immunization approach. Polyclonal antibodies have been raised against three proteins, namely, 16 kDa sperm protein, HSA, and Eppin. Singh et al. [126] observed that polyclonal antibody raised against a 16 kDa human sperm protein when administered passively in female mice resulted in reduction of fertility. It was likely that the antifertility effect was due to agglutinating nature of the antibody which inhibited the motility of spermatozoa. In another study, passive administration of antibodies to 80 kDa HSA in male and female rats resulted in agglutination of the spermatozoa with the loss of motility and impaired fertility [127]. Though still detailed toxicological evaluation needs to be conducted using nonhuman primate model (bonnet monkey). Also, passive immunization in mice with antibodies to a 26 kDa epididymal protein showed 89% reduction in fertility [128].

Further, monoclonal antibodies have been raised against human sperm and rabbit sperm, namely, MA-24 and mouse anti-rabbit sperm (MARS), respectively. Naz et al. [129] found that systemic intraperitoneal injection of MA-24 ascites fluid in mouse models reduced the percentage of fertilized ova (from 69% in untreated to 29% in treated), thereby reducing the fertility* in vivo* [130]. For determining the* in vivo* contraceptive efficacy of MARS, the antibodies were incubated* in vitro* with a defined volume of semen, followed by insemination of females. All the mAbs were found contraceptively effective [131].

## 3. Conclusion

In conclusion, it may be suggested that better understanding of the mechanisms of immunocontraception will lead to large scale applications of these methods in future. The review highlights that use of passive immunization approach is more feasible when compared with the concerns associated with the active immunization since data from antibody therapies including clinical trials in infectious diseases indicate that it is an exciting, practical, viable, and durable proposition ready for experimentation. Further, among all exploitation of sperm antigens as an immunocontraceptive agent after evaluating its feasibility and effectiveness can result in potent immunocontraception. Moreover, the generation of monoclonal antibodies in this league will act as “cherry” on top of the cake.
